# The School Career of Children With Hearing Loss in Different Primary Educational Settings—A Large Longitudinal Nationwide Study

**DOI:** 10.1093/deafed/enab008

**Published:** 2021-04-19

**Authors:** Tirza F K van der Straaten, Jeroen J Briaire, Evelien Dirks, Wim Soede, Carolien Rieffe, Johan H M Frijns

**Affiliations:** Department of Otorhinolaryngology and Head & Neck Surgery, Leiden University Medical Center, P.O. Box 9600, 2300 RC, Leiden, the Netherlands; Department of Otorhinolaryngology and Head & Neck Surgery, Leiden University Medical Center, P.O. Box 9600, 2300 RC, Leiden, the Netherlands; Dutch Foundation for the Deaf and Hard of Hearing Child, Lutmastraat 167, 1073 GX, Amsterdam, the Netherlands; Department of Otorhinolaryngology and Head & Neck Surgery, Leiden University Medical Center, P.O. Box 9600, 2300 RC, Leiden, the Netherlands; Department of Developmental Psychology, Leiden University, P.O. Box 9555, 2300 RB, Leiden, the Netherlands; Department of Psychology and Human Development, University College London, London WC1H 0AA, United Kingdom; Department of Otorhinolaryngology and Head & Neck Surgery, Leiden University Medical Center, P.O. Box 9600, 2300 RC, Leiden, the Netherlands; Leiden Institute for Brain and Cognition, Leiden University Medical Center, P.O. Box 9600, 2300 RC, Leiden, the Netherlands

## Abstract

Children with hearing loss (HL) are at risk for a lower educational achievement. This longitudinal study compared the school career of a nationwide Dutch cohort with and without HL based on descriptive data of the governmental authority Statistics Netherlands. From 2008 to 2018, 3,367,129 children, of whom 1,193 used cochlear implants (CIs) and 8,874 used hearing aids (HAs), were attending primary and/or secondary education. Sixty-one percent of children with HL attended mainstream and 31% special primary education. Compared to mainstreamed pupils without HL, mainstreamed pupils with HL achieved lower levels for language and mathematics in primary education but eventually attended comparable types of secondary education. Children with HL attending special primary education attained lower types of secondary education compared to mainstreamed peers with and without HL. These findings suggest that future educational (and as a result professional) attainment of a child with HL depends on the type of primary educational setting.

## Introduction

Early detection and hearing rehabilitation with hearing aids (HAs) and/or cochlear implants (CIs), family-centered early intervention, preschool treatment groups, and extra guidance at school have brought great enhancement for the development of children with hearing loss (HL) (Marschark & Spencer, 2011; Moeller et al., 2015; Yoshinaga-Itano, 2004). However, it remains unclear whether children with HL are nowadays able to reach their full potential in education, or that they are still at risk due to their HL (Dammeyer & Marschark, 2016; Illg et al., 2017; Nagle et al., 2016; Rydberg et al., 2009; Winn, 2007). Current knowledge regarding the school career of children with HL is built upon cohort studies that either examined the academic achievements during primary education (Harris et al., 2017; Khairi Md Daud et al., 2010; Qi & Mitchell, 2012; Wauters et al., 2006) or assessed the educational attainment of college students who were able to graduate from secondary or high school (Dammeyer & Marschark, 2016; Illg et al., 2017; Nagle et al., 2016; Rydberg et al., 2009; Winn, 2007). There is a lack of nationwide studies with a long-term follow-up investigating the type of secondary education of a large population with HL. Therefore, the present study examined the type of primary and secondary education in addition to the academic achievements of children with and without HL using a longitudinal design and a nationwide large sample in the Netherlands.

### Children with HL in special or mainstream primary education

Previously, in many Western countries children with HL were obliged to attend special schools for the deaf and hard of hearing (Mitchell & Karchmer, 2006). In these schools, children were surrounded by peers with HL and separated from their hearing peers. With current legislation in most Western countries (e.g., Education for all handicapped children act in the United States in 1975; Inclusive education in the Netherlands, August 2014), children with HL are encouraged to attend mainstream schools. As a result, respectively, 78% and 85% of the children with HL in the United States and Australia attend mainstream schools (Punch & Hyde, 2010; Shaver et al., 2014). Most of these children are HA-users as their relatively lower degree of HL allows them to attend mainstream schools at an early age (Shaver et al., 2014; Verhaert et al., 2008). Cohort studies examining the educational setting of children with CI reported a wide range of 38 to 64% children who fully or partially attended mainstream classes (Archbold et al., 2002; Christiansen & Leigh, 2002; Punch & Hyde, 2010). To our knowledge, the nationwide percentages of children with CIs or HAs in each educational setting have not yet been identified for other Western countries, such as the Netherlands.

There is still discussion concerning which children with HL will benefit from mainstream or special education (Stinson & Kluwin, 2012). The inclusive education policy of the Netherlands enables children with HL to have access to a mainstream school curriculum at a pace and in the same manner as it is taught to their hearing peers with an option to receive additional support of special education services. Literature has shown that children with HL in primary mainstream schools are likely to have higher academic achievements compared to children with HL in special schools (Marschark et al., 2015; Powers, 1999; Wauters et al., 2006). Still, mainstream education can be challenging for children with HL. Their auditory input is influenced by poor acoustics in large classrooms or background noise due to mumbling classmates which could lead to misunderstanding instructions and explanations of teachers. In other words, instructions in mainstream settings are not always communicatively accessible for children with HL.

Children with HL who have language and/or cognitive delays or special communication needs may lag behind even more in academic achievement due to their inability to keep up with mainstream education. Most of these children are therefore placed in special schools where support is provided in small groups or even on an individual level to allow for intensive guidance on their school performance. Other reasons, such as the severity of HL, additional handicaps, ethnicity, or the reliance on sign language (Israelite et al., 2002; Karchmer et al., 1982; Knoors & Vervloed, 2012; Rydberg et al., 2009; Shaver et al., 2014), may influence the decision whether the child will benefit from special (for the deaf or hard of hearing) or mainstream education. Due to these reasons, children with HL in special education are often supported by sign language and individually evaluated instead of taking standardized academic achievement tests.

### Essential subjects in primary education

To continue in secondary education, children need to acquire essential scholastic skills. Among the diverse subjects in school, language and mathematics are two main subjects of standardized achievement tests in primary education. Commencing with a language delay due to a deprived auditory input can continue to affect the development of language and mathematics. Learning to read is one of the biggest challenges children with HL face in school (Geers & Hayes, 2011; Trybus & Karchmer, 1977; Worsfold et al., 2010). Previous studies found that children with HL in general hover between a third or fourth-grade reading level (Qi & Mitchell, 2012) or that roughly 4% of deaf students within special education read on an age-appropriate level (Wauters et al., 2006). Further on in their school career, “learning to read” moves to “reading to learn” which gives children with reading deficits even more challenges to obtain an educational degree (Walter & Dirmyer, 2013). Factors such as aided audibility, the degree of HL, age at identification, and age at cochlear implantation, which influence language development in children with HL, have also been found to affect reading development (Archbold et al., 2008; McCann et al., 2009; McCreery et al., 2015; Moeller et al., 2007, 2015).

Children with HL seem to score lower in mathematical assignments (Gottardis et al., 2011; Nagle et al., 2016; Sarant et al., 2015; Swanwick et al., 2005; Traxler, 2000), although this has been less often subject of research compared to language and reading. Difficulties are found in number comparisons, calculation, counting, number facts, numeral language, mathematical concepts, measurement, story problems, multiplication, and fractions (Ansell & Pagliaro, 2006; Frostad & Ahlberg, 1999; Kritzer, 2009; Leybaert & Van Cutsem, 2002). Mathematics often requires reading comprehension and understanding of specific linguistic math terms such as conditionals, comparatives, and inferentials (Traxler, 2000). Hence, mathematical achievement tends to co-vary with reading ability (Edwards et al., 2013; Mukari et al., 2007). Solving mathematical exercises is therefore expected to be extra challenging for children with HL who have reading and language difficulties. Previous studies did not find differences between CI and HA-users in their mathematical achievement (Bull et al., 2018; Marschark et al., 2015), which might result from the heterogeneity within the HL population (Convertino et al., 2009) and the sample sizes (Bull et al., 2018). Current knowledge of the degree of mathematical skills in children with HAs and CIs is still limited and further research is required.

### Secondary education and individuals with HL

To the best of our knowledge, no research to date has yet examined which type of secondary education adolescents with HL attend. It is known that college students with HL have a higher risk of obtaining lower educational attainment compared to their hearing peers (Dammeyer & Marschark, 2016; Illg et al., 2017; Nagle et al., 2016; Rydberg et al., 2009; Winn, 2007). It is even estimated that only about 40% of the pupils with HL obtain their secondary school diploma (Idstad & Engdahl, 2019; Powers, 2003; Teasdale & Sorensen, 2007). Lower educational attainment might eventually lead to a higher chance of unemployment later in life (Dammeyer et al., 2019; Winn, 2007).

To date, all available conclusions about the educational achievements of individuals with HL are mostly based on small cross-sectional studies with a high probability of selection bias. Some studies were conducted on larger samples, but these large-scale studies are weighted heavily toward either profoundly deaf students in special settings (Illg et al., 2017; Qi & Mitchell, 2012) or college students with mild/moderate HL in mainstream settings (Dammeyer et al., 2019; Hendar & O’Neill, 2016; Idstad & Engdahl, 2019; Teasdale & Sorensen, 2007). A longitudinal nationwide large-scale study that covers the whole population of children with HL from primary school years to adolescence is still lacking.

### Educational system in the Netherlands

In the Netherlands, education starts at age 4 and includes 8 years in primary education. Schooling is compulsory from the age of 5 to 16 years. Most Dutch children with HL start with specialized preschool treatment groups to support their language and communication skills. Thereafter, parents and professionals decide whether the child will benefit from special education (for the deaf or hard of hearing) or they can keep up with mainstream education. This decision is often based on the level of language, communication, and social skills of the child. There is little empirical evidence which of these two types of educational settings would enable individuals with HL best to reach their full potential (Shaver et al., 2014; Stinson & Kluwin, 2012).

Dutch children are obliged to complete a final test in their last year of primary mainstream education (e.g., Cito, IEP, Route 8) (Lubbe, 2007). It covers compulsory subjects such as language (e.g., reading) and mathematics, but also includes geography, history, and subjects about nature. The standard score of this test estimates the type of secondary education that the child could potentially obtain. Unlike other countries such as the United States and France, secondary education in the Netherlands is uniquely divided into four types from the first year onwards. Based on the outcomes of this final test, Dutch children are divided in either low or intermediate prevocational, general secondary, or preuniversity education (Hakkenes & de Wijs, 2012). This Dutch system (with varied types of secondary education) aims to focus on the potential an individual has for attending and successfully accomplishing secondary education (Dutch Ministry of Education Culture and Science, 2006).

### Present study

**Figure 1.**
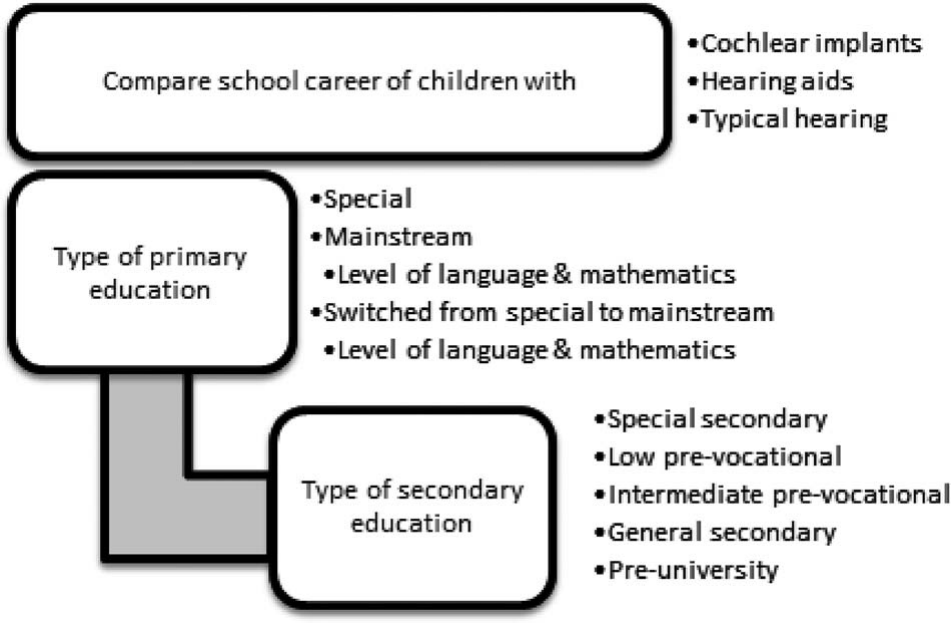
Research model of this study.

The main aim of this longitudinal nationwide study was to unravel the school career of children with HL in different educational settings in a large population. First, the distribution of children with HL in either special or mainstream primary education was studied. Second, children with and without HL were compared on their grades for language and mathematics obtained in primary mainstream education based on the outcomes of a national standardized test (Cito). Moreover, the impact of switching from special to mainstream education on the school career was examined by dividing the mainstream group into children who always attended mainstream education and children who switched to mainstream education. Third, the type of secondary education of adolescents with HL was examined and compared to their typical hearing (TH) peers, taking their primary educational settings (i.e., mainstream, special, and switched from special to mainstream education) into account. Figure 1 illustrates an overview of the research questions. Due to small sample sizes within the HL population, previous research could not identify if the use of either HAs or CIs was related to differences in academic achievement or in the type of education (Bull et al., 2018; Convertino et al., 2009). This study was based on data from the governmental authority Statistics Netherlands which enabled us to design a large-scale longitudinal and nationwide study, and to compare individuals with either HAs or CIs to the Dutch hearing population. A longitudinal follow-up through different school years allowed us to monitor these children with HL from primary to secondary education. An intrinsic limitation of using this kind of nationwide generic collected data is that other HL-related background information is lacking, such as the degree and etiology of HL or the age at detection of HL and intervention.

## Materials & Methods

### Study design

This longitudinal retrospective study was designed using existing nonpublic microdata from Statistics Netherlands (www.cbs.nl). This third trusted party has nationwide data available with strict regulations on privacy and data anonymity. For example, the day of birth was censored and data that involved 10 individuals or fewer was not to be disclosed. Consequently, some categories needed to be combined to ensure the privacy of small groups. Under these conditions, microdata are accessible for statistical and scientific research without additional approval of an ethics committee. For further information: microdata@cbs.nl. We selected all children who were born between 1995 and 2013. The billed medical care, which is based on the combination of diagnosis and treatment, was available between 2013 and 2017 and was used to define if a child was using a CI or HA. These children received medical care in a hospital at least once during the period from 2013 to 2017. This is because in the Netherlands, children with CIs receive follow-up examinations every 6 months until the age of 11 years, thereafter follow-up changes to once a year. Children with permanent HL and HAs receive follow-up examination every year until the age of 11 years, and afterwards changes to once every 2 years. By using these data, we recognized that we were unable to identify the children with a mild or profound HL who did not use HAs or CIs (estimation of .7% missing that has sensorineural HL). Each child living in the Netherlands is obliged to follow education from the age of 5 to 16 years. Every Dutch child should therefore be enrolled in one of the Dutch schools. Data on the type of primary education that each child was attending were used to track whether they attended mainstream or special schools during the school years 2008 till 2018. Standardized academic achievement scores were available for the school years 2006 to 2018. Type of secondary education was available for the school years 2007 till 2018. Demographic data on every child, including sex, ethnicity, parental educational attainment, and household income were also collected.

### Study population

The study population consisted of 4,087,877 children, of which 1,283 used CIs and 9,677 used HAs. These numbers corresponded with the national registry of pediatric cochlear implantations in the Netherlands (https://www.opciweb.nl/ci-centra/ci-centra-in-nederland/aantal-implantaties-in-nederland-t-m-2017/). Children with HL were separated into three groups by type of educational setting during their primary school years: children who only attended mainstream schools, children who (eventually) attended special schools, and children who had switched from special to mainstream schools (characteristics in Table 1). Some children were not registered in public primary schools which resulted in 710,681 (17%) children having missing data regarding their educational settings (due to their age, additional disabilities, emigration, or other unknown reasons). There were significantly more girls in the group with HL who continually attended mainstream schools compared to the group of children with HL in special schools. This female preponderance was also apparent when we compared children with typical hearing (TH) in mainstream and special education. Additional nonauditory disabilities were most prevalent within children with HAs who attended special education compared to all other groups (most of them had down syndrome [8%] and/or behavioral problems [3.6%]). Children who (eventually) attended mainstream schools were more often autochthonous (native Dutch), had parents with significantly higher educational attainment and a higher household income compared to children who attended special schools. This difference was found in both groups with and without HL.

**Table 1:** Characteristics of children with typical hearing (TH), with cochlear implants (CIs), and with hearing aids (HAs)

		TH	CI			HA		
		(a) *n* = 3,165,074	Mainstream (b) *n* = 259	Switched (c) *n* = 242	Special (d) *n* = 692	Mainstream (e) *n* = 5,830	Switched (f) *n* = 316	Special (g) *n* = 2,728
Sex *n* %	Boys	***1,585,542*** ***50,1%*** ^***e***^	114 44,0%	127 52,5%	*366* *52,9%^b,e^*	2,748 47,1%	*183* *57,9%^a,b,e^*	*1,565* *57,4%^a,b,d,e^*
	Girls	***1,579,532*** ***49,9%*** ^***f,g***^	*145* *56,0%^d,f,g^*	*115* *47,5%^d,f,g^*	*326* *47,1%^g^*	*3,082* *52,9%^a,d,f,g^*	133 42,1%	1,163 42,6%
Additional nonauditory disability *n %*	No	***3,105,633*** ***98,1%***^***d,e,f,g***^	*249* *96,1%^g^*	*232* *95,9%^g^*	*664* *96,0%^g^*	*5,626* *96,5%^g^*	*300* *94,9%^g^*	2,378 87,2%
	Yes	59,441 1,9%	10 3,9%	10 4,1%	*28* *4,0%^a^*	*204* *3,5%^a^*	*16* *5,1%^a^*	*350* *12,8%^a,b,c,d,e,f^*
Highest education of mother *n* %	Missing	*979,809* *31,0%^c^*	*85* *32,8%^c^*	50 20,7%	*211* *30,5%^c^*	*1,788* *30,7%^c^*	*97* *30,7%^c^*	*844* *30,9%^c^*
	Primary or secondary education	*487,106* *15,4%^b,c,e^*	26 10,0%	23 9,5%	*201* *29,1%^a,b,c,e,f,g^*	784 13,4%	*52* *16,5%^b,c^*	*677* *24,8%^a,b,c,e,f^*
	Vocational education	*672,424* *21,2%^e^*	54 20,9%	56 23,1%	142 20,5%	1,123 19,3%	62 19,6%	*675* *24,7%^a,d,e,f^*
	Polytechnics or University	*1,025,735* *32,4%^d,g^*	*94* *36,3%^d,g^*	*113* *46,7%^a,b,d,e,f,g^*	138 19,9%	*2,135* *36,6%^a,d,g^*	*105* *33,2%^d,g^*	532 19,5%
Gross income of household *n* %	Unknown	3,175 0,1%	1 0,4%	1 0,4%	2 0,3%	7 0,1%	1 0,3%	3 0,1%
	€0–15,000	*726,471* *23,0%^e,f^*	53 20,5%	44 18,2%	*199* *28,8%^a,b,c,e,f,g^*	1,166 20,0%	54 17,1%	*681* *25,0%^a,c,e,f^*
	€15–45,000	*317,828* *10,0%^b,e^*	15 5,8%	*30* *12,4%^b^*	*119* *17,2%^a,b,e,f^*	519 8,9%	*35* *11,1%^b^*	*426* *15,6%^a,b,e,f^*
	€45–60,000	280,305 8,9%	20 7,7%	28 11,6%	*78* *11,3%^a,e^*	512 8,8%	35 11,1%	*309* *11,3%^a,e^*
	>€60,000	*1,837,295* *58,0%^d,g^*	*170* *65,6%^a,d,g^*	*139* *57,4%^d,g^*	294 42,5%	*3,626* *62,2%^a,d,g^*	*191* *60,4%^d,g^*	*1,309* *48,0%^d^*
Ethnic origin *n %*	non-native	*626,474* *19,8%^b,c,e^*	29 11,2%	26 10,7%	*236* *34,1%^a,b,c,e,f,g^*	778 13,3%	*57* *18,0%^b,c,e^*	*713* *26,1%^a,b,c,e,f^*
	Native Dutch	*2,538,600* *80,2%^d,g^*	*230* *88,8%^a,d,f,g^*	*216* *89,3%^a,d,f,g^*	456 65,9%	*5,052* *86,7%^a,d,f,g^*	*259* *82,0%^d,g^*	*2,015* *73,9%^d^*

For each significant pair, the key of the category with the smaller column proportion appears in the category with the larger column proportion (*p* < .001).

### Standardized academic achievement test at the end of primary mainstream education

The national standardized academic achievement test (developed by the National Board of Tests and Examinations and the Central Institute for Test Development [Cito]) was used to indicate the performance level of children at the end of primary education (https://www.cito.com/) (Lubbe, 2007). This exam, often referred to as Cito or the central final test, is conducted by roughly two-thirds of the schools in the Netherlands (schools decide which test they use [e.g., Cito, IEP, route 8]). It indirectly indicates intelligence, motivation, concentration, and drives to learn and has a well-documented reliability (Hakkenes & de Wijs, 2012; Lek, 2020). The test consists of multiple-choice questions covering the obligatory subjects language and mathematics (world orientation such as geography, history, and nature are not mandatory). Questions covering language involve reading comprehension, summarizing, writing skills, and language cultivation (spelling, grammar, and vocabulary). Mathematical tasks cover measurements, geometry, time, money, fractions, and ratios. These are regular questions that require reading comprehension. Dutch schools are obliged to provide test accommodations (e.g., extra examination time, extra support with sign language, pictures, or assistive listening devices) for students with extra needs such as dyslexia or hearing problems. The raw scores were converted to percentile scores for further analyses.

### Types of secondary education

Secondary education in the Netherlands starts after primary education at around the age of 12 years and is compulsory until the age of 16. It ranges from 4 to 6 years depending on the type of education. When entering secondary education, pupils are divided into one of the four different types of education: low prevocational (basic and general occupation-oriented education or in Dutch VMBO-basis/praktijk), intermediate prevocational (combination of general and theoretical occupation-oriented education or VMBO-gemengd/theoretisch), general secondary (HAVO), and preuniversity (VWO). Each stream demands increasing intellectual and scholastic abilities (Hakkenes & de Wijs, 2012). Pupils can switch upward or downward between the types of secondary education depending on their academic achievement. After secondary education, pupils can attend further optional higher education: vocational education for graduates of low or intermediate prevocational education, polytechnics for graduates of general secondary education, or university for graduates of preuniversity education (www.epnuffic.nl). Special secondary education in the Netherlands provides education that is mainly focused on acquiring skills for the labor market or finding daytime activities with the opportunity (not mandatory) for acquiring an educational degree (often low prevocational education) (Dutch Ministry of Education Culture and Science, 2014). The schools thus can provide adjustments to the curriculum based on the developmental capabilities and educational needs of each pupil.

### Statistical analysis

We used all nonpublic microdata from Statistics Netherlands that was available in February 2020. The date of birth was censored, and therefore age was calculated based on the year of birth. Children with HL were divided into three groups based on their type of educational setting during primary school years (2008–2018): children who only attended mainstream schools, children who (eventually) attended special schools, and children who had switched from special to mainstream schools. To examine a potential increase in the proportion of children with HL in mainstream schools, the different school years (2008–2018) were compared with dependent samples *t*-tests. Descriptive statistics were used for the baseline characteristics between groups. To compare the standardized test scores of children with CIs and HAs to children with TH in mainstream education, one-way ANOVA and independent samples *t*-tests were performed for the percentile scores of language and mathematics. Furthermore, a chi-square (χ2) test was carried out to examine the proportion of pupils attending each type of secondary education and whether the distributions differed between the different groups. Due to the privacy regulations of Statistics Netherlands some categories have been combined to ensure the privacy of small groups (e.g., merging general secondary and preuniversity education). Statistical analyses were performed with IBM SPSS Statistics 23.0 software package.

## Results

### Distribution in primary education

**Figure 2.**
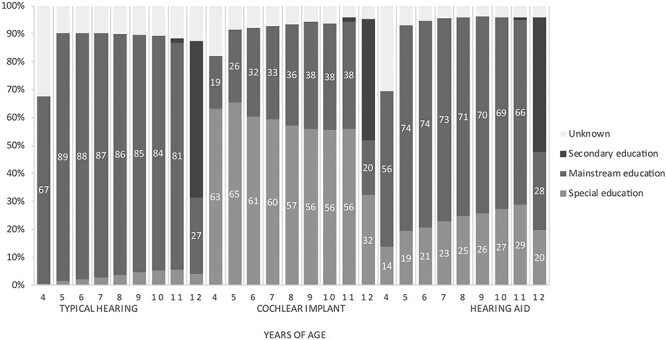
Distribution of children within primary educational settings in percentages.

The type of education during primary school years is shown in Figure 2 for children with TH (n = 3,165,074), CIs (n = 1,193), and HAs (n = 8,874). Considering all primary school years together, 61% of children with HL (eventually) attended mainstream schools and 31% special schools. Sixty-four percent and 28% of the HA-users and 39% and 54% of the CI-users attended mainstream and special schools, respectively. The type of primary educational setting of the remaining 8% (CI n = 90 and HA n = 803) of children with HL was unknown or they were already in secondary education. Compared to the TH population, fewer children with CIs and HAs attended mainstream schools (*p* < .05). Within the HL population, children with HAs attended mainstream education more frequently compared to children with CIs *p* < .05).

At age 5, 65% of the children with CIs were in special schools and 26% in mainstream schools. Eventually at age 11, respectively, 56% and 38% of the children with CIs attended special and mainstream schools. This means that the proportion of children with CIs in mainstream schools significantly increased from 5 to 11 years of age (*p* < .05). Within the total group of CI-users, 20% of the children stayed in mainstream education from the start, 19% switched from special to mainstream education, 50% stayed in special education, and 4% switched from mainstream to special education.

The number of children with HAs in mainstream schools significantly decreased from 5 to 11 years of age (*p* < .05). At age 5, 19% of the children with HAs were in special schools and 74% were in mainstream schools. At age 11, respectively, 29% and 66% of the children with HAs attended special and mainstream schools. Within the total group of HA-users, 8% of the children switched from mainstream to special education, 20% stayed in special education, 3% of the children with HAs were able to switch from special to mainstream education, and 60% stayed in mainstream education from the start.

Subsequently, the proportion of children with HL in mainstream settings per schoolyear was examined over time (2008 to 2018). Only the percentage of children with CIs in mainstream settings considerably increased over time (Figure in supplements). There was a clear distinction, however not statistically different (*p* > .05), between children with CIs who were born before and in 2005 and onwards: 6-year-old children attended mainstream education more often from the schoolyear 2011 than before and 10-year-old children attended less often mainstream education before the schoolyear 2015 than after 2015 and onwards. Thus, children with CIs born from 2005 and onwards appeared to attend mainstream schools more often compared to the ones born before 2005.

### Language and mathematics at the end of primary mainstream education

**Figure 3.**
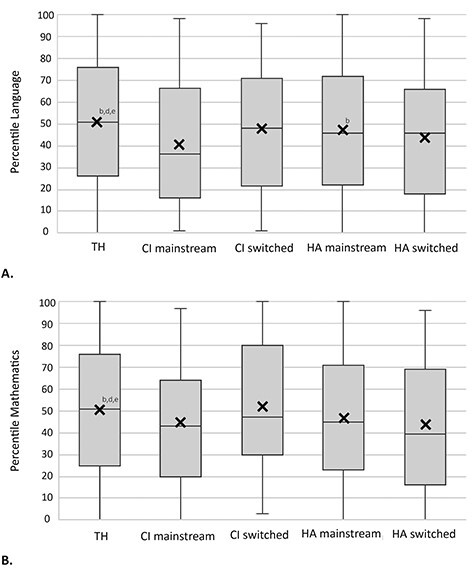
Boxplots of the level of language (A) and mathematics (B) in percentile scores of children with typical hearing (TH), cochlear implants (CI), and hearing aids (HA). The cross represents the mean and the horizontal line the median of each group. For each significant pair, the key of the smaller category appears in the category with a larger mean (*p* < .05).

In line with the fact that two-thirds of the schools use Cito in the Netherlands (Lubbe, 2007), we found that around two-thirds of the mainstreamed children in the data (who were old enough) completed this standardized test (70% [n = 1,345,287] of the children with TH, respectively 63% [n = 74] and 70% [n = 120] of the children with CIs who switched to or continuously attended mainstream education, and respectively 59% [n = 110] and 67% [n = 2,543] of the children with HAs who switched to or continuously attended mainstream education). Only a negligible number of children in special schools took the standardized test, which impeded us from examining their language and mathematics scores. The average score of language and mathematics was higher for children with TH compared to children with HAs and CIs (Figure 3). Yet, children with CIs who switched from special to mainstream schools had comparable levels of language and mathematics as their hearing peers. Among those who continuously attended mainstream education, children with HAs outperformed children with CIs with respect to language, but not with respect to mathematics.

In Table 2 the children are divided in quartiles to indicate if their percentile scores fell into a below-average (25^th^ percentile and below), average (25–75^th^ percentile), or above average (75^th^ percentile and above) group. Approximately 60–70% of the children with HL performed average or above average on language and mathematics. Regarding language specifically, more mainstreamed children with CIs and HAs performed below average compared to children with TH (*p* < .05). Concerning mathematics, more children with HAs performed below average compared to children with TH and children with CIs who switched to mainstream education (*p* < .05). Strikingly, more children with CIs who switched to mainstream education performed above average on mathematics compared to their peers with CIs and HAs who always attended mainstream education. In addition, post hoc analysis showed a positive correlation between language and mathematics (*r* = .501–.682, *p* < .001) in all groups.

**Table 2:** Percentage of children who performed on average, below, or above average on language and mathematics

	TH	CI		HA	
	(A) *n* = 1,345,287	Mainstream (B) *n* = 120	Switched (C) *n* = 74	Mainstream (D) *n* = 2,543	Switched (E) *n* = 110
*Language* Below (0-25^th^) Average (25-75^th^) Above (75-100^th^)	25,0% 49,3% *25,7%^,d,e^*	*37,7%^a^* 43,0% 19,3%	32,1% 50,0% 17,9%	*29,4%^a^* 49,0% 21,6%	33,3% 51,7% 14,9%
*Mathematics* Below (0-25^th^) Average (25-75^th^) Above (75-100^th^)	25,2% 48,9% *25,9%^b,d^*	29,2% 53,3% 17,5%	21,6% 47,3% *31,1%^b,d^*	*28,9%^a^* >49,9% 21,2%	*37,3%^a,c^* 41,8% 20,9%

*Bold* = significantly larger proportion than the category mentioned in superscript (*p* < .05).

TH = typical hearing; CI = cochlear implants; HA: hearing aids

### Type of secondary education

**Figure 4.**
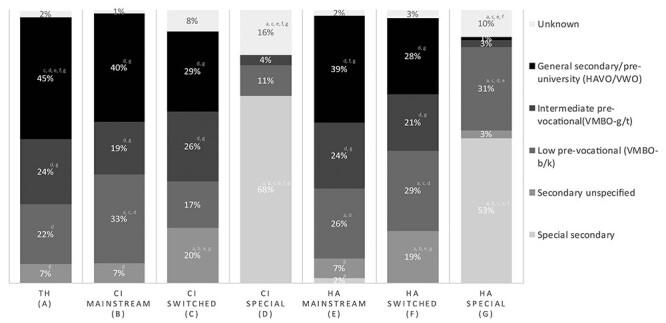
Distribution of the type of secondary education within adolescents (age 13 to 18 years) with typical hearing (TH; n = 1,130,777), cochlear implants (CI; respectively n = 83, 89, 263), or with hearing aids (HA; respectively n = 2,392, 136, 1,092) who attended mainstream primary education only, who switched from special to mainstream primary education, and who attended special primary education. For each significant pair, the key of the category with the smaller column proportion appears in the category with the larger column proportion (*p* < .05).

The different types of secondary education was examined between adolescents with TH (n = 1,130,777), CIs (mainstream n = 83; switched n = 89; special n = 263), or HAs (mainstream n = 2,392; switched n = 136; special n = 1,092) who were 13 to 18 years of age between the school years 2007–2018 (Figure 4). Adolescents with CIs and HAs, who finished their primary education in mainstream schools, followed roughly the same distribution in secondary education as their hearing peers. However, adolescents with CIs attended low prevocational education (Figure 4.B) and unspecified secondary education (level of secondary education not yet determined) (Figure 4.C) significantly more often compared to TH peers (*p* < .05). There were also significantly more adolescents with HAs in special, low prevocational, or unspecified secondary education compared to their hearing peers (*p* < .05; Figure 4E and F). On the contrary, adolescents with TH attended more often general secondary or preuniversity education than adolescents with HL (both children with CIs and HAs; *p* < .05). Post hoc analysis showed a positive correlation between language scores, mathematical scores and the type of secondary education (language *r* = .309–.623, *p* < .021–.001; mathematics *r* = .309–.523, *p* < .07–.001). This indicated that pupils who obtained higher levels of language and mathematics at the end of mainstream primary education attended types of secondary education with higher intellectual challenge.

After primary education in special schools, adolescents with CIs and HAs attended special secondary education significantly more often and intermediate prevocational, general secondary, or preuniversity significantly less often compared to all other groups (Figure 4D and G). Regarding low prevocational education, more HA-users attended this educational level compared to hearing pupils, CI-users who switched to mainstream education and who stayed in special education, and HA-users who stayed in mainstream education (Figure 4.G).

## Discussion

This longitudinal retrospective study is to our knowledge the first to compare the school career of a nationwide cohort with HL and their hearing peers from school-age to adolescence. Overall, 61% of the children with HL attended mainstream and 31% special primary education. However, we found that more HA-users attended mainstream education compared to CI-users (64% versus 39%). The majority of children with HL who attended mainstream education reached average or above average levels of language and mathematics similar to their hearing peers. After continuously attending mainstream education, children with HAs outperformed children with CIs regarding language. Yet, children with CIs who switched from special to mainstream primary education achieved comparable levels of language and mathematics as their hearing peers, though children with HAs were unable to reach that score.

This difference between children with CIs and HAs disappeared during secondary education. Adolescents with HL who (eventually) attended mainstream primary education went to similar types of secondary education compared to their hearing peers. Only low prevocational, unspecified, or special secondary education were more often attended, and general secondary or preuniversity education were less often attended by adolescents with HL. Most notably, individuals with HL who have been only in special primary education attended lower levels of secondary education than their mainstreamed peers with and without HL. Many of the adolescents from special primary schools continued their school careers in specialized education. This study, therefore, revealed that not all children with HL, but mainly the children who finished their primary education in special settings are expected to obtain a lower educational achievement after graduating from their secondary school.

### Mainstream primary education and standardized achievement outcomes

In line with the literature where children with HL tend to underachieve on scholastic examinations (Edwards et al., 2013; Geers & Hayes, 2011; Marschark et al., 2015; Mukari et al., 2007; Pagliaro & Kritzer, 2013; Spencer & Marschark, 2010; Trybus & Karchmer, 1977), this study also found lower mean levels of language and mathematics in children with HAs and CIs compared to hearing children. However, a majority of children with HL performed above or on average when we evaluated their language and mathematical scores based on the percentile quartile they were in. The reason for this inconsistency might be twofold. First, this study was able to omit selection bias by using a standardized test in a nationwide population with HL who attended mainstream and not special education. Second, the division of below-, on-, and above-average might be better for evaluating this heterogeneous group of mainstreamed children with HL whose scores covered a broad spectrum. It is likely that the children within the below-average group compromised the average score of the complete group with HL. Moreover, post hoc analyses showed a positive correlation between language and mathematics. This indicates that children who had lower levels of language also underperformed on mathematical tasks and vice versa. This might support the fact that mathematical tasks require an understanding of specific linguistic terms or reading comprehension (Edwards et al., 2013; Mukari et al., 2007).

Thus, this study found that a majority of children with HL in mainstream primary education could keep up with their hearing peers as they showed comparable academic achievements. These are promising results for the growing population of children with HL in mainstream settings. The increase of children with CIs in mainstream settings over time might be a result of early detection of congenital HL, which enabled early awareness and rehabilitation through family-centered early intervention. The newborn hearing screening was completely implemented in the Netherlands in 2005 (Korver et al., 2013), which ensures early development of language and communication (Yoshinaga-Itano, 2004). It is expected that early detection of HL and intervention will continue to enable children with HL to transfer to mainstream education and obtain average educational achievements.

Furthermore, this study corroborated that the educational chances in secondary education of children with an auditory disability are good as long as they can attend mainstream education, even with the different conditions of a secondary school in mind. Acoustics, listening effort, social–emotional inclusion, or the time-frame at which their (HL) identity is developed are key-factors that children with HL have to deal with besides attaining adequate educational achievements in secondary education (Brice & Strauss, 2016; Israelite et al., 2002; Kent & Smith, 2006; Rich et al., 2013; Spencer et al., 2012).

### Switching from special to mainstream primary education

After switching from special to mainstream primary education, children with CIs were able to achieve similar levels of language and mathematics as their hearing peers. Possibly, by attending special education in the early years prepared these children to keep up with the curriculum in mainstream schools. Alternatively, these CI-users might have been assigned to special education while in hindsight they would have had even better opportunities to reach their full potential if their initial placement would have been in mainstream education. However, this did not apply to children with HAs. Instead, children with HAs who made the step from special to mainstream primary education lagged behind on language and mathematics compared to their peers without HL. This could be related to the fact that children with HAs usually switch at an earlier age compared to children with CIs. Children with HAs tend to speak relatively well and often have successful interactions with others which might leave their difficulties unnoticed (Tomblin et al., 2015). Yet, the discrepancy between children with CIs and HAs was not maintained in secondary education. This finding may encourage parents and teachers of children with CIs to consider the transition to mainstream education despite their slight delay in language and mathematics as they will eventually attend similar types of secondary education. Thus, not the level of academic performance but the primary educational setting of children with HL was related to the educational achievement later in life.

### Special primary education

In total, 54% of all children with CIs and 28% of the children with HAs attended special primary education and did not switch to mainstream education. A subset of these children could have attended some hours or days in mainstream education which is common practice in the Netherlands. However, we were not able to distinguish the amount of participation in our dataset, but only identified in which educational setting each child was registered. This might explain our lower epidemiological numbers compared to the United States and Australia (of which 78% and 85% of the children with HL attend mainstream schools) (Punch & Hyde, 2010; Shaver et al., 2014). It would be interesting to further investigate the reasons for such a large group of children with HL (more specifically children with CIs) staying in special education, including the reasons other than their level of (spoken) language and communication skills. Multiple factors, such as sign language, the severity of HL, additional handicaps (despite low percentages), ethnicity, and so on, have possibly contributed to the fact that these children with HL were assigned to specialized education with more individual support (Israelite et al., 2002; Karchmer et al., 1982; Knoors & Vervloed, 2012; Rydberg et al., 2009; Shaver et al., 2014). It is also possible that a switch to mainstream education was withheld by parents and/or teachers. As a result, these children with HL continued in specialized settings during their adolescence and attained a lower educational achievement.

### Strengths and limitations

No studies to date have examined the level of mathematical skills in such a large group of children with HL. Besides, this study is the first to examine the educational attainment during adolescence as most previous studies were conducted in college years (Dammeyer & Marschark, 2016; Marschark et al., 2015). The longitudinal format made it also possible to delve into the impact of primary educational settings on the educational achievement of adolescents with HL. With the available data of Statistics Netherlands, we could examine a large sample of children with CIs and HAs in the Netherlands without selection biases. However, a limitation of using the national data of Statistics Netherlands was a lack of additional background information, such as the type and degree of HL, the age at detection of HL and at intervention, the reason for educational placement, and the level of support children with HL receive at their school. The standardized achievement test was used in two-third of the mainstream schools, implying that we had missing data of one-third of the total mainstreamed population with HL in the Netherlands. This was however not a consequence of a selection bias, but a decision made within the mainstream schools whether or not they used Cito or other standardized tests. Additionally, a lack of standardized achievement tests in special education prevented us from investigating the levels of language and mathematics of children with HL in specialized settings. This will change as the government of the Netherlands has recently made standardized tests in special primary education obligatory. Adding the grades of these children in special education to the mainstreamed population with HL will most likely decrease the overall mean levels of language and mathematics of the group with HL. Furthermore, the choice for educational setting does not only depend on the academic performance. Some adolescents might lack great interest in obtaining high educational achievements, but would rather have peers that are similar to them. Future studies should therefore consider the social perspectives of each educational setting and include children from more recent cohorts with CIs and HAs that have benefitted from the ongoing innovations in the field of hearing technology and interventions.

## Conclusions

The majority of children with HL were able to keep up with mainstream education and attended similar types of secondary education as their hearing peers. In these mainstream settings, children with CIs did not differ or performed better on academic achievements compared to children with HAs. After finishing primary education in special settings, children with HL attended more often special secondary education than their mainstreamed peers with and without HL. On the basis of these findings, extra guidance and precautions should be made in special education to inform caregivers and teachers about future perspectives. This enables shared decision making regarding the best educational setting for children with HL in order to reach their full potential. Moreover, mainstream schools, with the additional support from the Dutch government, need to be more inclusive for children with HL, especially for the ones with CIs.

## Funding

This work was supported by Stichting het Heinsius-Houbolt Fonds.

## Conflicts of interest

None.
